# Assessment of Knowledge and Attitude of Medical Practitioners Towards Pediatric Dental Care in Raichur District, Karnataka, India-A Survey

**DOI:** 10.5005/jp-journals-10005-1543

**Published:** 2018-10-01

**Authors:** Dhanu G Rao, Shamama Sheereen, Raghavendra Havale, Shrutha S Prakasha, Manva M Zulfikar

**Affiliations:** 1 Professor, Department of Pedodontics and Preventive Dentistry, AME’s Dental College and Hospital, Bengaluru, Karnataka, India; 2 Postgraduate Student, Department of Pedodontics and Preventive Dentistry, AME’s Dental College and Hospital, Bengaluru, Karnataka, India; 3 Professor, Department of Pedodontics and Preventive Dentistry, AME’s Dental College and Hospital, Bengaluru, Karnataka, India; 4 Reader, Department of Pedodontics and Preventive Dentistry, AME’s Dental College and Hospital, Bengaluru, Karnataka, India; 5 Senior Lecturer, Department of Conservative Dentistry and Endodontics, AME’s Dental College and Hospital, Bengaluru, Karnataka, India

**Keywords:** Attitude, Knowledge, Medical practitioner.

## Abstract

**Aim:**

To determine the knowledge and attitude of medical practitioners towards dental care of children in Raichur district.

**Materials and methods:**

The present study is a cross-sectional survey conducted among the medical practitioners of Raichur district. The study was conducted on 300 practitioners, randomly selected. The data pertaining to their knowledge and attitude about oral health was gathered using a self-administered questionnaire. Data were analyzed using descriptive studies.

**Results:**

Among the study subjects, 32.7% of the respondents could recognize the precancerous and cancerous lesions in the oral cavity. A total of 65.3% considered that dental caries is not infectious. About 52.7% of the physicians thought that scaling causes tooth sensitivity and only 22.7% knew that tooth brushing should be initiated after the eruption of the first milk tooth.

**Conclusion:**

Medical practitioners had a moderate knowledge and attitude towards pediatric dental care.

**How to cite this article:** Rao DG, Sheereen S, Havale R, Prakasha SS, Zulfikar MM. Assessment of Knowledge and Attitude of Medical Practitioners Towards Pediatric Dental Care in Raichur District, Karnataka, India-A Survey. Int J Clin Pediatr Dent, 2018;11(5):375-381.

## INTRODUCTION

One of the significant challenges posed by the dental fraternity is the maintenance of a population with good oral health. The dental disorder is not just a minor disease of the gums and teeth, but a disease of the body that happens to commence in the oral cavity.^1^ Medical practitioners especially the pediatrician and gynecologist are the first to get in touch with the child since birth and has a responsibility to educate the parents. Thus, the physicians are in the supreme position to align parents regarding the prevention of oral diseases and impart knowledge leading to healthy oral environment.^2^ Thereby, it is preferable for the physicians to possess the fundamental dental understanding to unmask signs and symptoms of dental diseases. Very few studies have collected data concerning the dental knowledge of physicians. The present study seeks to assess the knowledge and attitude of medical practitioners towards child dental care.

## MATERIALS AND METHODS

A cross-sectional survey was conducted among the medical practitioners of Raichur district, Karnataka. A simple random sampling was done. Sample size of 300 medical practitioners was decided. Data regarding their knowledge and attitude towards dental health was gathered using a self-administered questionnaire comprising of 25 close-ended questions divided into two sections. One section contained the questions to assess the knowledge and the other section for attitude. The investigator approached each practitioner personally and distributed the questionnaire. It was informed that responses would remain anonymous. At the end of the questionnaire queries about their personal details like name, gender, and medical specialty were recorded. The filled questionnaire was immediately collected within 20 minutes and analyzed.

### Inclusion Criteria

The practitioners should have registered in the Karnataka state medical council.

- They should be practicing in Raichur district.
- They should either be pursuing postgraduation or should have completed their postgraduate course.

### Scoring Criteria

The scores were assessed as follows:

- *< 50%:* Poor
- *50 to 75%:* Moderate
- *> 75%:* Good

### Statistical Analyses

Descriptive statistics such as frequency and percentage was used to present the data. Data analysis was done by using Microsoft Excel.

## RESULTS

Responses of the study subjects on polar questions based on their knowledge and attitude towards pediatric dental care are tabulated in Table 1. The results showed that 67.3% of the practitioners knew that pediatric dentistry is a specialty. About 32.7% of the respondents could recognize the precancerous and cancerous lesions in the oral cavity. Almost half of the practitioners were not aware that dental sealants will prevent caries.

**Graph 1:**
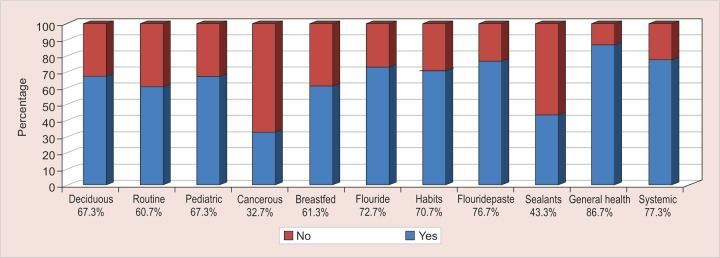
Response of study subjects on polar questions based on dental knowledge

**Graph 2:**
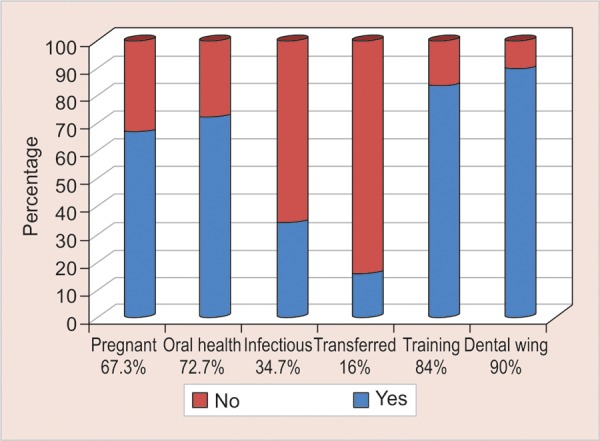
Response of study subjects on polar questions based on dental attitude

A total of 67.3% were aware that pregnant women need a dental check-up, 65.3% considered that dental caries is not infectious and only 16% knew it is transmissible from mother to child. The graphical representation of the polar question on knowledge is illustrated in Graph 1 and attitude in Graph 2.

**Table 1:** **Table 1:** Polar questions based on knowledge and attitude

*Polar questions based on knowledge*		*Yes (%)*		*No (%)*	
Is treating deciduous teeth important?		67.3		32.7	
Consider oral examination as a part of routine general check up?		60.7		39.3	
Aware of the existence of pediatric dentistry		67.3		32.7	
as an exclusive specialty?					
Able to recognize the precancerous and		32.7		67.3	
cancerous lesions in the oral cavity?					
Relation between long term breastfeeding/		61.3		38.7	
bottle feeding on dentition?					
Familiar with the effect of fluoride on oral health?		72.7		27.3	
Oral habits affect the dentition of the child?		70.7		29.3	
Aware of fluoride toothpaste in preventing		76.7		23.3	
dental caries?					
Aware of fluoride sealants?		43.3		56.7	
Relation between oral health and general health		86.7		13.3	
Can untreated dental disease lead to systemic complications?		77.3		22.7	
Polar questions based on attitude		yes (%)		no (%)	
Pregnant women need dental check up?		67.3		32.7	
Educate your patients about importance of oral health?		72.7		27.3	
Is dental caries an infectious disease?		34.7		65.3	
Is dental caries transferred from mother to child?		16.0		84.0	
Interested to receive oral health care training?		84.0		16.0	
Is dental wing necessary in rural areas?		90.0		10.0	

**Graph 3:**
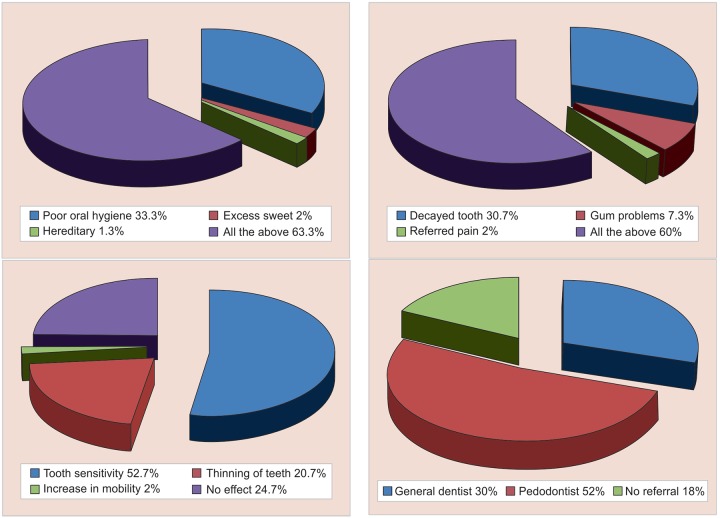
Response of study subjects on MCQs based on dental knowledge

**Graph 4:**
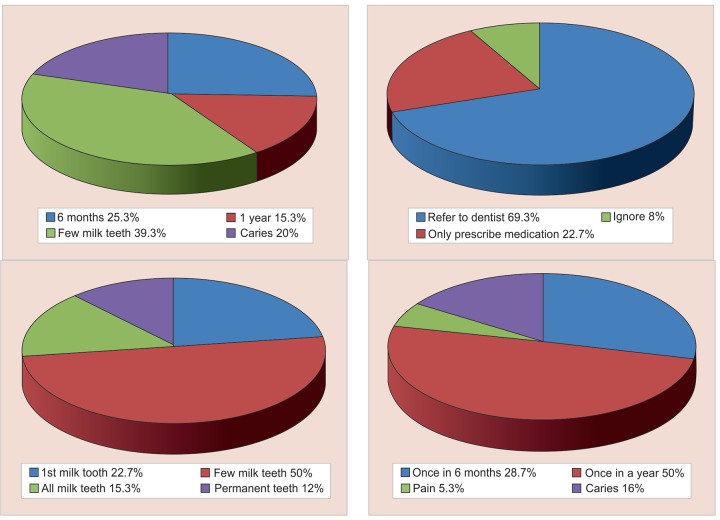
Response of study subjects on MCQs based on dental attitude

Table 2 displays the response on multiple choice questions (MCQs). The results indicate that about 52.7% of physicians thought that scaling causes tooth sensitivity. Only 22.7% of respondents knew that tooth brushing should be initiated after the eruption of the first milk tooth and 28.75% answered that frequency of dental visits is once in 6 months. Graphs 3 and 4 summarize the response of the MCQs on knowledge and attitude respectively.

The results of the study revealed that the knowledge and attitude of medical practitioners towards pediatric dental health was moderate.

## DISCUSSION

Medical practitioners are considered the primary bond between dentists and children. Dental diseases can be treated at early stages if the physicians examine the oral cavity regularly. This study emphasizes the crucial role that the physicians can play to enhance oral public health.

**Table 2:** **Table 2:** Multiple choice questions based on knowledge and attitude

*MCQs based on knowledge*			
Common causes of dental problems?		Percentage	
Poor oral hygiene		33.3	
Eating excess sweet		2.0	
Hereditary		1.3	
All the above		63.3	
Common cause of pain in the oro facial region?		Percentage	
Decayed tooth		30.7	
Gum problems		7.3	
Referred pain from other parts of the body		2.0	
All the above		60.0	
Adverse effect of scaling on teeth?		Percentage	
Tooth sensitivity		52.7	
Thinning of teeth		20.7	
Increase in mobility		2.0	
No effect		24.7	
In your clinic if you come across a child with dental problems, whom would you refer the child to?		Percentage	
General dentist		30.0	
Pedodontist		52.0	
No referral		18.0	
*MCQs based on attitude*			
First dental visit for a child?		Percentage	
When the baby is 6 months		25.3	
When the baby is one year		15.3	
After eruption of a few milk teeth		39.3	
Dental caries present		20.0	
What do you do if a patient with dental abscess reports to your clinic?		Percentage	
Refer the patient to dentist		69.3	
Only prescribe medications		22.7	
Ignore		8.0	
Commencement of tooth brushing?		Percentage	
After the eruption of first milk tooth		22.7	
After eruption of a few milk teeth		50.0	
After eruption of all milk teeth		15.3	
Only after eruption of permanent teeth		12.0	
Frequency of dental visits?		Percentage	
Once in 6 months		28.7	
Once in a year		50.0	
Only if in pain		5.3	
When dental caries is noticed		16.0	

In the present study with regards to dental knowledge, 67.3% of medical practitioners knew the importance of treating deciduous teeth and 60.7% of the respondents examined oral cavity routinely. However, Al-Hussyeen et al. reported that almost half of pediatricians did not routinely include dentition in their examinations.^3^

Only 32.7% of the doctors were able to recognize the precancerous and cancerous lesions in the oral cavity which is in correlation with the study conducted by Umesh et al.^4^ Participants stated that lack of knowledge regarding early signs and symptoms was a major hindrance to the diagnosis of oral pre-cancer and early cancer.^5^

Among the surveyed doctors 61.3% were familiar with the harmful effects of long-term breastfeeding/ bottle feeding. Appropriate breastfeeding is considered the best feeding method for infants. But, certain feeding habits like nocturnal breastfeeding, at will-breast feeding, and weaning delayed beyond the age of 2 years could all harm the dentition.^6-8^ Sabbagh et al. found the majority of pediatricians (81.3%) were familiar with the harmful effects of night breastfeeding.^6^ In a previous study by Murthy et al. more than 50% of doctors felt that only bottle fed children get ECC. But there is evidence to show that infants who sleep with the mother and nurse throughout the night are prone to increased caries risk.^9^^11^

With regards to fluoride, 72.7% of the practitioners were aware of the effect of fluoride on oral health, awareness of fluoride toothpaste existed among 76.7% of the respondents, and the knowledge of fluoride sealants was prevalent amongst nearly half of the participants (43.3 %). As investigated by Poornima et al. the pediatricians’ knowledge of caries prevention and potentials of fissure sealants were found to be limited, considering that 64% of pediatricians did not even know what fissure sealants were.^2^ Similar results were also reported by previous studies.^3,12,13^

Oral habits include digit sucking, pacifier sucking, lip sucking and biting, nail-biting, bruxism, self-injurious habits, mouth breathing, and tongue thrust. Among the respondents, 70.7% were aware that oral habits affect the dentition of the child. The physicians can provide the parents with information regarding the consequences of a habit.

Among the investigating population, 86.7% were well acquainted of the relation between oral and general health, while the knowledge was slightly higher among the doctors in a study conducted by Ashok et al.^1^ The harmful role of gum/periodontal disease in many serious and life-threatening diseases is evident from recent studies. For example, periodontal disease is associated with coronary heart disease; diabetes; low pre-term birth weights; respiratory disease; improper digestion; osteoporosis; stress the immune system; lower resistance to infections; and minimizes life expectancy.^14-18^ In the present study, most of them (77.3%) have rightly answered that untreated dental disease can lead to systemic complications.

Pregnancy is an essential phase in a woman’s life, and good oral health is essential for the mother and the baby as well. Although prenatal education is considered the key to dental care of the infant, proper instructions regarding oral health are not delivered to the pregnant women.^19^ Among the study subjects, 67.3 % suggested the need for a dental checkup in pregnant women whereas Srinidhi et al. published a study in which a majority (91%) of the practitioners were aware of its significance.^20^

The present study showed that more than half (72.7%) of the doctors alert their patients about the importance of oral health. Among the participants, 65.3% were unaware that dental caries is a disease which is infectious. Only 16% of them knew that cavity-causing bacteria could be transmitted from the mother to children, which is also cited in the pediatric literature.^21^ *S. mutans* can be transmitted by Vertical transmission (from mother to child) and/or horizontal transmission (between members of a group e.g. family members or students in a classroom). Poor maternal oral hygiene, dietary habits, child-rearing habits, sharing food and utensils, breastfeeding and sleeping beside the mother, were all significantly associated with colonization of *S. mutans.* The major source from which infants acquire *S. mutans* is their mothers.^22-25^

Since majority were not trained with respect to oral health aspects, 84% of the respondents showed their interest to receive oral health care training and 90% recommended for a dental wing in primary health centers.

When questioned about the common causes of dental problems, 33.3% of the doctors thought it could be because of poor oral hygiene, 2% because of excess sweet and 1.3% said heredity, while 63.3% believed that dental problems are multifactorial.

On investigation about the common cause of pain in the orofacial region, 30.7% said decayed tooth could be the etiology, 7.3% opted for gum problems, and 2% of the practitioner’s reflection was on referred pain from other parts of the body while 60% answered for all of the above factors.

When asked to reflect their views on scaling, only 24.7% have answered the correct option that scaling does not have any adverse effect on teeth. While 52.7% said scaling results in tooth sensitivity, 20.7% responded thinning of teeth, 2.0% said the increase in mobility.

Among the participants, 67.3% of the doctors were aware of the existence of pediatric dentistry as an exclusive specialty. Only 52% of practitioners referred children to a pedodontist while 30% refer to a general dentist. In a study conducted by Poornima et al.^2^ around 86% of pediatricians referred children with oral disease/dental caries to pedodontist.

When investigated about the first dental visit, 25.3% of the practitioners suggested the parents for the first dental visit of the child at 6 months of age, 15.3% when the baby is one year and 39.3% after the eruption of a few milk teeth. Early visits to the dentist are recommended since it facilitates preventive measures, early diagnosis, alignment regarding proper diet and nutrition, oral hygiene instructions and prevention of non-nutritive sucking habit.^26-28^

According to the American Academy of Pediatric Dentistry (AAPD) guidelines, tooth brushing should be initiated after the eruption of first milk tooth and the AAPD recommends for a biannual dental visit.^29^ In the present study, half of the doctors thought tooth brushing should be initiated after the eruption of a few milk teeth and only 22.7% of them rightly counseled the parents for brushing after the eruption of first milk tooth. Murthy and Mohandas reported 33.3% of physicians recommended commencement of tooth brushing after the eruption of first milk tooth.^9^

Regarding the frequency of dental visits, only 28.7% of the respondents suggested their patients visit the dentist once in six months while 50% thought once in a year. Chandra et al.^30^ revealed that regular visit of once in six months was suggested by 86.3% of practitioners which is high when compared with the present study.

In Raichur, people lack awareness pertaining to the oral health. One of the reasons may be that Raichur district comes under Hyderabad Karnataka region which is considered as the underdeveloped region with low socioeconomic status and poor education. This makes the physician’s role critical. Medical practitioners are considered as social health reformers in delivering knowledge to the common masses.

## LIMITATION

The limitation of the survey was the inability to use open-ended questions to probe the participant’s responses to a greater extent.

## CONCLUSION

The study indicates that the practitioners had moderate knowledge and attitude towards pediatric dental health. They feel they have a crucial role to be played in the promotion of oral health, but since they are not well acquainted with oral health issues and have not received much dental health education, it makes it difficult for them to promote prevention of dental diseases. Thereby appropriate steps have to be taken for a better understanding of the dental related issues.

## RECOMMENDATIONS

- Collaboration between dentists and medical practitioners for implementation of oral health programs and seminars.
- To inspire the establishment of associate clinics which includes providing medical and dental services under one roof.
- Publish the preventive dentistry articles in medical journals.
- Imparting oral health education in the form of brochures, posters, etc.
- Establish a dental home along with the medical home.

These measures help in establishing a good rapport between the dentist and medical practitioners and helps to impart oral health knowledge to the medical doctors.
